# Molecular basis of non-deletional HPFH in Thailand and identification of two novel mutations at the binding sites of CCAAT and GATA-1 transcription factors

**DOI:** 10.1038/s41598-023-39173-8

**Published:** 2023-07-24

**Authors:** Kritsada Singha, Anupong Pansuwan, Mattanee Chewasateanchai, Goonnapa Fucharoen, Supan Fucharoen

**Affiliations:** 1 Centre for Research and Development of Medical Diagnostic Laboratories, Faculty of Associated Medical Sciences, Khon Kaen University, Khon Kaen, 40002 Thailand; 2 Faculty of Medicine, Mahasarakham University, Kantharawichai, Mahasarakham Thailand; 3 Regional Health Promotion Center 7, Ministry of Public Health, Khon Kaen, Thailand

**Keywords:** Genetics, Molecular biology, Diseases, Molecular medicine

## Abstract

High Hb F determinants are genetic defects associated with increased expression of hemoglobin F in adult life, classified as deletional and non-deletional forms. We report the first description of non-deletional hereditary persistence of fetal hemoglobin (HFPH) in Thailand. Study was done on 388 subjects suspected of non-deletional HPFH with elevated Hb F expression. Mutations in the ^G^γ- and ^A^γ-globin genes were examined by DNA analysis and rapid diagnosis of HPFH mutations were developed by PCR-based methods. Twenty subjects with five different mutations were identified including three known mutations, − 202 ^A^γ (C>T) (n = 3), − 196 ^A^γ (C>T) (n = 3), and − 158 ^A^γ (C>T) (n = 12), and two novel mutations, − 117 ^A^γ (G>C) (n = 1) and − 530 ^G^γ (A>G) (n = 1). Interaction of the − 117 ^A^γ (G>C) and Hb E (HBB:c.79G>A) resulted in elevation of Hb F to the level of 13.5%. Two plain heterozygous subjects with − 530 ^G^γ (A>G) had marginally elevated Hb F with 1.9% and 3.0%, whereas the proband with homozygous − 530 ^G^γ (A>G) had elevated Hb F of 11.5%. Functional prediction indicated that the − 117 ^A^γ (G>C) and − 530 ^G^γ (A>G) mutations dramatically alter the binding of transcription factors to respective γ-globin gene promotors, especially the CCAAT and GATA-1 transcription factors. Diverse heterogeneity of non-deletional HFPH with both known and new mutations, and complex interactions of them with other forms of thalassemia are encountered in Thai population.

## Introduction

The inherited disorders of hemoglobin (Hb) or hemoglobinopathies are the commonest human monogenic diseases, found in about 7% of the world population. These can be broadly classified into 3 groups, structural Hb variants, thalassemia and hereditary persistence of fetal hemoglobin (HPFH)^1–3^. In Thailand, high prevalence of hemoglobinopathies has been documented including 20–30% of α-thalassemia, 3–9% of β-thalassemia, 20–30% of Hb E (HBB:c.79G>A), 1–8% of Hb Constant Spring (HBA2:c.427T>C) and Hb Pakse′ (HBA2:c.429A>T), and other structural Hb variants and high Hb F determinants. These lead to diverse heterogeneity and complex thalassemia syndromes in the country^4,5^. Accurate diagnosis of these hemoglobinopathies is important for promoting appropriate management, genetic counseling, and a prevention and control program of thalassemia in the region.

During early childhood, the level of Hb F normally declines to less than 1–2% of total Hb. The level of Hb F is therefore less than 1–2% in normal adult. High Hb F determinants are a group of genetic defects associated with increased expression of Hb F in adult life. The conditions include δβ-thalassemia, γδβ-thalassemia and hereditary persistence of fetal hemoglobin (HPFH). While δβ-thalassemia and γδβ-thalassemia are usually associated with elevated Hb F and hypochromic microcytic red blood cell indices, HPFH is associated with higher Hb F expression with normal red blood cells. Homozygous or compound heterozygous for δβ-thalassemia and β-thalassemia may have clinical phenotype of non-transfusion-dependent thalassemia or transfusion-dependent thalassemia. In contrast, homozygous HPFH or compound heterozygous HPFH and β-thalassemia usually have no clinical symptom. However, these clinical phenotypes are generally overlapped which renders their differentiation in routine setting difficult unless DNA analysis is performed^5–7^. Since co-inheritance of these high Hb F determinants in β-thalassemia disease has an ameliorating effect on the disease severity, identification of them in routine practice is important. At the molecular level, they could be classified into deletional and non-deletional high Hb F determinants. While the former involves several large DNA deletions removing δ- and β-globin genes, the latter is usually caused by point mutations on ^G^γ- or ^A^γ-globin gene promoters affecting the binding of related transcription factors^1,3,5^.

In Thailand, several deletional forms of δβ-thalassemia and HPFH have been documented. These included HPFH-6 (79.3 kb deletion) (NG_000007.3:g.45595_124872), δβ^0^-thalassemia (12.6 kb deletion) (NG_000007.3:g.64383_76994), Indian deletion-inversion ^G^γ(^A^γδβ)^0^-thalassemia (NG_000007.3:g.48400_49245del;49246_64567inv;64568_72051del), Chinese ^A^γδβ^0^-thalassemia (78.9 kb deletion) (NG_000007.3:g.48795_127698del), δβ^0^-thalassemia (11.3 kb deletion) (NG_000007.3:g.60045_71313delinsTACATTAAGAGATACCTTAATG), Siriraj ^A^γδβ^0^-thalassemia (~ 118 kb deletion) (AC104389.8:g.52507_165744), Thai deletion-inversion-insertion ^A^γδβ^0^-thalassemia (NG_000007.3:g.47449_165744del;168412_168590invins;insAAGAAGA) and Vietnamese/SEA HPFH (27 kb deletion) (NG_000007.3:g.64384_76993del)^5–10^. However, non-deletional form of HFPH has rarely been described. This study provides for the first time the molecular description of non-deletional HFPH in Thailand including known and novel mutations affecting the CCAAT and GATA-1 transcription factors binding.

## Materials and methods

### Subjects and hematological analyses

This study was conducted in accordance with the Declaration of Helsinki and ethical approval of this study protocol was obtained from the Institutional Review Board of Khon Kaen University, Thailand (HE652154). Left-over blood specimens of 388 subjects suspected of non-deletional HPFH were selectively recruited at our routine thalassemia diagnostic service at Khon Kaen University, Thailand. They were all negative for deletional forms of high Hb F determinants previously found in Thailand^5–10^. These included subjects with normal β-globin gene but Hb F ≥ 1.0% (n = 81), Hb E heterozygotes with Hb F ≥ 1.0% (n = 287), β-thalassemia carriers with Hb F>10% (n = 6), and homozygous Hb E with Hb F>20% (n = 14). For comparison, normal individuals with Hb F < 1.0% (n = 60) were also analyzed. Hematological data is routinely recorded on standard automated blood cell counter. Hb analysis is performed using automated capillary electrophoresis system (Capillarys 2 Flex Piercing; Sebia, Lisses, France) or automated high-performance liquid chromatography (HPLC) (VARIANT™; Bio-Rad Laboratories, Hercules, CA, USA).

### Routine DNA analysis

Common α-thalassemia (--^SEA^, --^THAI^, -α^3.7^, -α^4.2^, Hb Constant Spring, and Hb Pakse′), β-thalassemia, deletional high Hb F determinants, and Krüppel-like factor 1 (KLF1) mutations found in Thai population were identified by PCR-based methods as described previously^5,9,11–14^. ^G^γ- and ^A^γ-globin promoter mutations were examined by DNA sequencing on ABI PRISM™ 3730 XL analyzer (Applied Biosystems, Foster City, CA, USA) using primers F35 and γ5^15^, and F22^9^ and γ35^16^. Sequences of all primers used in this study are listed in the Supplementary Table S1.

### Identification of the − 158 (C>T) ^G^γ- and − 158 (C>T) ^A^γ-globin gene promoters

The PCR-restriction fragment length polymorphism (PCR–RFLP) assay for detection of − 158 ^G^γ- globin promoter (C>T) (HBG2:c.− 211C>T) and − 158 ^A^γ-globin promoter (C>T) (HBG1:c.− 211C>T) was developed as shown in Supplementary Fig. S1. Selective amplification of the ^G^γ- and ^A^γ-globin gene promoters was done using primers γ4^15^ and γ5 (577 bp in length), and F22 & γ5 (639 bp in length), respectively. With this assay, the amplified product was digested to completion with *Xmn*I restriction enzyme (5′-GAANN^▼^NNTTC-3′) (New England Biolabs, Beverly, MA, USA). After digestion, the 577 bp of − 158 ^G^γ-globin promoter with T allele [*Xmn*I (+)] was digested into two fragments with 402 bp and 175 bp in lengths, and the 639 bp fragment for the − 158 ^A^γ- globin promoter with T allele [*Xmn*I (+)] was digested into two fragments with 464 bp and 175 bp in lengths. The − 158 ^G^γ- and ^A^γ-globin promoters with C allele [*Xmn*I (−)] remain undigested^9,15,16^.

### Identification of − 202 ^A^γ (C>T), − 196 ^A^γ (C>T), − 117 ^A^γ (G>C), and − 530 ^G^γ (A>G)

**Figure 1:**
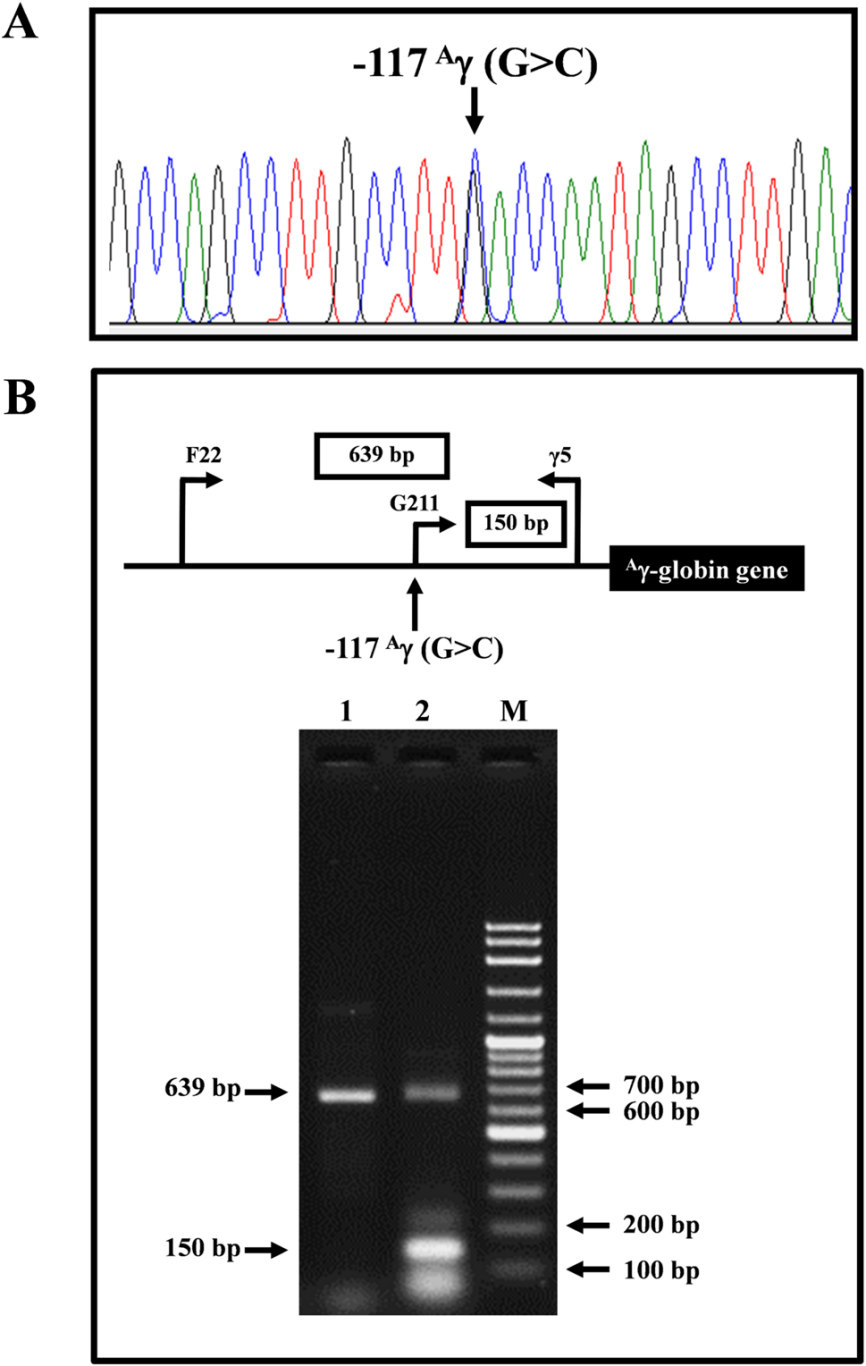
DNA sequencing profiles of heterozygous for − 117 ^A^γ (G>C) (**A**) and identification of the − 117 ^A^γ (G>C) by allele-specific PCR assay (**B**). M represents the VC 100 bp plus DNA Ladder (Vivantis Technologies Sdn Bhd). Lanes 1 and 2 are subjects with negative and positive for the mutation, respectively.

**Figure 2:**
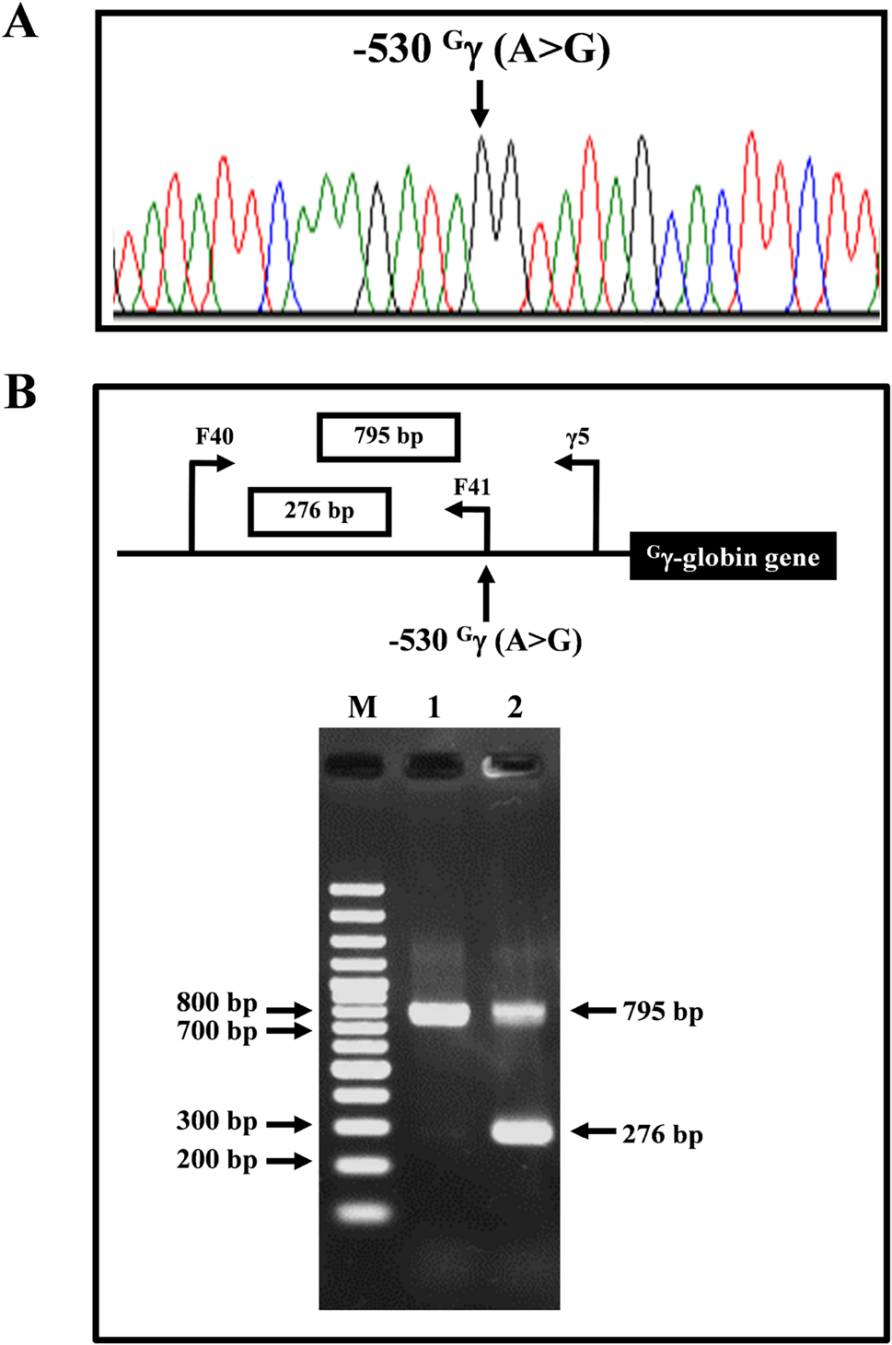
DNA sequencing profiles of homozygous − 530 ^G^γ (A>G) (**A**) and identification of the − 530 ^G^γ (A>G) by allele-specific PCR assay (**B**). M represents the VC 100 bp plus DNA Ladder. Lanes 1 and 2 are subjects with negative and positive for the mutation, respectively.

Allele-specific PCR assays were developed for rapid identification of four non-deletional HPFH mutations including − 202 ^A^γ-globin (C>T) (HBG1:c.− 255C>T) (Supplementary Fig. S2), − 196 ^A^γ-globin (C>T) (HBG1:c.− 249C>T) (Supplementary Fig. S3), − 117 ^A^γ-globin (G>C) (HBG1:c.− 170G>C) (Fig. 1), and − 530 ^G^γ-globin (A>G) (HBG2:c − 583A>G) (Fig. 2). Common primer pairs (F22 & γ5) were used to produce the 639 bp amplified internal control fragment for − 202 ^A^γ-globin (C>T), − 196 ^A^γ-globin (C>T), and − 117 ^A^γ-globin (G>C) mutations. Similarly, primers (F40 & γ5) were used to produce the 795 bp internal control fragment for − 530 ^G^γ-globin (A>G) mutation. Specific primer pairs (F38 & F22), (F39 & F22), (G211 & γ5), and (F41 & F40) were used to generate specific fragments of 444, 450, 150, and 276 bps in length for the − 202 ^A^γ-globin (C>T), − 196 ^A^γ-globin (C>T), − 117 ^A^γ-globin (G>C), and − 530 ^G^γ-globin (A>G), respectively. The PCR mixture (50 μl) contains 50–200 ng genomic DNA, 30 pmol each primer, 200 μM dNTPs and 1 unit *Taq* DNA polymerase (New England Biolab, Inc., USA) in 10 mM Tris-HCl (pH 8.3), 50 mM KCl, 0.01% gelatin and 3 mM MgCl_2_. The amplification reaction was carried out on a T-Personal Thermocycler (Biometra; GmbH, Gottingen, Germany). A total of 30 cycles after initial heating at 94 °C for 3 min was performed under the following PCR condition: 93 °C 30 s, 62 °C 30 s, and 72 °C for 1 min. The amplified PCR product was separated on 2.0% agarose gel electrophoresis and visualized under UV light after ethidium bromide staining.

### β-Globin gene haplotype analysis

Seven polymorphic restriction sites on β-globin gene cluster including ε-*Hinc*II, ^G^γ-*Hind*III, ^A^γ-*Hind*III, ψβ-*Hinc*II, 3′ψβ-*Hinc*II, β-*Ava*II and 3′β-*BamH*I were determined using PCR–RFLP assays as described previously^17^.

### Prediction on the effects of novel γ-globin gene promoter mutations on the binding of transcription factors

The online TFBIND software (https://tfbind.hgc.jp/)^18^ was used to compare the binding affinity of transcription factors to the promoters of γ-globin genes with wild-type sequences and their mutant counterparts including − 117 ^A^γ (G>A), − 117 ^A^γ (G>C), − 114 ^A^γ (C>T), − 530 ^G^γ (A>G), and − 533 to − 529 (-ATAAG) mutations.

## Results

Among 388 subjects recruited DNA sequencing of ^G^γ- and ^A^γ-globin genes was selectively carried out in 177 subjects with Hb F>5.0%. Fourteen subjects (7.9%) with five mutations in the γ-globin gene promoters were identified including three known mutations, − 202 ^A^γ (C>T) (n = 3), − 196 ^A^γ (C>T) (n = 3), and − 158 ^A^γ (C>T) (n = 6), and two novel mutations, − 117 ^A^γ (G>C) (n = 1) and − 530 ^G^γ (A>G) (n = 1). All mutations but the − 530 ^G^γ (A>G) were identified in heterozygotic form. Rapid PCR diagnosis based on PCR–RFLP assays (Supplementary Fig. S1) and allele specific PCR assays (Figs. 1, 2, Supplementary Figs. S2 and S3) for detection of these non-deletional HPFH mutations were developed. The assays were then used to screen the remaining 211 subjects with Hb F 1.0–5.0% as well as 60 normal individuals with Hb F < 1.0%. With this screening, we further identified six subjects (2.8%) with the − 158 ^A^γ (C>T) mutation on PCR–RFLP based assay. No mutation was detected in 60 normal subjects with Hb F < 1.0%. Therefore, a total of 20 of 388 (5.1%) recruited subjects were found to carry HPFH mutations. These included in total − 202 ^A^γ (C>T) (n = 3), − 196 ^A^γ (C>T) (n = 3), − 158 ^A^γ (C>T) (n = 12), − 117 ^A^γ (G>C) (n = 1) and − 530 ^G^γ (A>G) (n = 1). As shown in Table 1, among these 20 subjects, eight subjects had normal β-globin gene, 11 subjects were also carriers of Hb E. The remaining subject (Table 1, case no 17) with − 158 ^A^γ (C>T) HPFH mutation and Hb F 27.1% was a boy (2-yr-old) who was found to be a carrier of β^0^-thalassemia (β^17(AAG>TAG)^, HBB:c.52A>T) and α^+^-thalassemia (3.7 kb deletion). This boy was therefore a triple heterozygote for ^A^γ-HPFH, β^0^-thalassemia and α^+^-thalassemia, a hitherto undescribed complex condition. Extended family analysis indicated that the − 158 ^A^γ (C>T) mutation was detected in *trans* to the β^0^-thalassemia gene. He had Hb A_2_ of 4.3%, still within diagnostic range for a β-thalassemia trait.

**Table 1:** Hematological parameters, Hb analysis, and genotypes of 20 Thai subjects with non-deletional HPFH.

Case no.	Sex/age (year)	RBC (10^12^/L)	Hb (g/dL)	HCT (%)	MCV (fL)	MCH (pg)	MCHC (g/dL)	RDW (%)	Hb type	Hb E + A_2_ (%)	Hb A_2_ (%)	Hb F (%)	− 158 ^G^γ *Xmn*I	KLF1	α-genotype	β-genotype
− 202 ^A^γ (C>T)																
1	M/Adult	5.7	14.8	44.6	77.7	26.0	33.2	na	EFA	30.5	–	8.7	+/−	Normal	αα/αα	β^E^/β^A^
2	F/Adult	5.1	12.1	35.8	70.1	23.7	33.8	na	EFA	21.9	3.9	9.4	+/−	H299D	αα/αα	β^E^/β^A^
3	F/Adult	4.4	10.8	33.3	76.0	24.8	32.5	na	EFA	20.5	3.4	11.7	+/−	T334R	α^CS^α/αα	β^E^/β^A^
	Total	5.1 ± 0.7	12.5 ± 2.0	37.9 ± 5.9	74.6 ± 4.0	28.8 ± 1.2	33.2 ± 0.7					9.9 ± 1.6				
− 196 ^A^γ (C>T)																
4	M/35	4.5	13.8	41.0	90.3	30.5	33.7	13.4	A_2_FA	**–**	2.6	12.2	−/−	Normal	αα/αα	β^A^/β^A^
5	M/31	6.0	17.0	52.7	88.0	28.6	32.4	12.9	A_2_FA	**–**	1.6	15.1	−/−	Normal	-α^3.7^/αα	β^A^/β^A^
6	M/Adult	5.8	15.7	49.7	86.2	27.2	32.6	13.6	A_2_FA	**–**	1.5	15.7	+/−	Normal	αα/αα	β^A^/β^A^
	Total	5.4 ± 0.8	15.5 ± 1.6	47.8 ± 6.1	88.2 ± 2.1	28.8 ± 1.7	32.9 ± 0.7	13.3 ± 0.4				14.3 ± 1.9				
− 158 ^A^γ (C>T)																
7	M/29	5.7	15.9	46.6	81.2	27.7	34.1	13.8	EA	25.0	–	2.1	+/+	Normal	-α^3.7^/αα	β^E^/β^A^
8	M/Adult	5.3	15.4	46.7	89.0	29.2	33.0	11.3	EA	29.6	3.8	3.2	+/+	Normal	αα/αα	β^E^/β^A^
9	F/Adult	4.5	10.7	34.4	76.7	23.9	31.1	13.9	A_2_A	**–**	3.3	3.5	+/−	Normal	-α^3.7^/α^CS^α	β^A^/β^A^
10	F/Adult	na	na	na	79.3	26.2	33.0	na	A_2_A	**–**	3.0	4.2	+/−	Normal	α^CS^α/αα	β^A^/β^A^
11	F/Adult	4.8	12.0	35.4	74.2	25.2	33.9	na	ConSpA_2_A	**–**	2.3	4.4	+/−	Normal	α^CS^α/αα	β^A^/β^A^
12	M/Adult	4.6	12.6	38.0	82.9	27.3	33.0	12.5	EA	30.5	3.7	4.6	+/+	Normal	αα/αα	β^E^/β^A^
13	F/28	4.9	13.3	38.5	77.9	26.9	34.5	13.8	EFA	28.5	3.8	7.2	+/−	Normal	αα/αα	β^E^/β^A^
14	F/Adult	4.5	11.7	34.2	75.8	25.9	34.2	na	EFA	25.3	**–**	7.0	+/+	Normal	αα/αα	β^E^/β^A^
15	F/Adult	3.4	10.8	31.8	78.5	26.7	34.0	na	EFA	27.8	**–**	11.0	+/−	Normal	αα/αα	β^E^/β^A^
16	F/Adult	4.3	11.6	35.4	83.0	27.4	32.9	13.3	EFA	29.1	3.8	11.5	+/+	Normal	αα/αα	β^E^/β^A^
17	M/2	5.9	12.0	36.0	60.9	20.3	33.3	18.7	A_2_FA	**–**	4.3	27.1	+/−	Normal	-α^3.7^/αα	β^17(AAG>TAG)^/β^A^
18	M/Adult*	5.3	13.8	42.0	80.0	26.0	32.9	na	A_2_FA	**–**	2.6	7.6	+/+	G176Afs*179	-α^3.7^/αα	β^A^/β^A^
	Total	4.8 ± 0.7	12.7 ± 1.7	38.1 ± 5.0	78.3 ± 6.7	26.1 ± 2.2	33.3 ± 0.9	13.9 ± 2.3				7.8 ± 6.8				
− 117 ^A^γ (G>C)																
19	M/Adult	na	na	na	71.8	na	na	na	EFA	30.3	1.7	13.5	+/−	Normal	αα/αα	β^E^/β^A^
− 530 ^G^γ (A>G)																
20	M/33*	5.1	13.4	39.9	78.4	26.3	33.6	12.9	A_2_FA	**–**	2.6	11.5	−/−	Normal	αα/αα	β^A^/β^A^

*Found as homozygote, na: not available.

No mutation in both γ-globin genes was identified in subjects with homozygous Hb E with unusually high Hb F level. Further screening for the KLF1 mutations previously described in Thai population showed that 3 of these 20 subjects with HPFH (case no. 2, 3 & 18) carried the KLF1 mutations i.e., H299D (NG_013087.1:g.6869C>G) (n = 1), T334R (NG_013087.1:g.7231C>G) (n = 1) and G176Afs*179 (NG_013087.1:g.6493_6499dupCGGCGCC) (n = 1). α^+^-Thalassemia was detected in 8 of 20 HPFH subjects with relatively decreased Hb F levels as compared to those with the same HPFH mutations but without α-thalassemia. The overall hematological parameters, Hb analysis results as well as α- and β-globin genotypes of these 20 non-deletional HPFH were summarized in Table 1.

**Figure 3:**
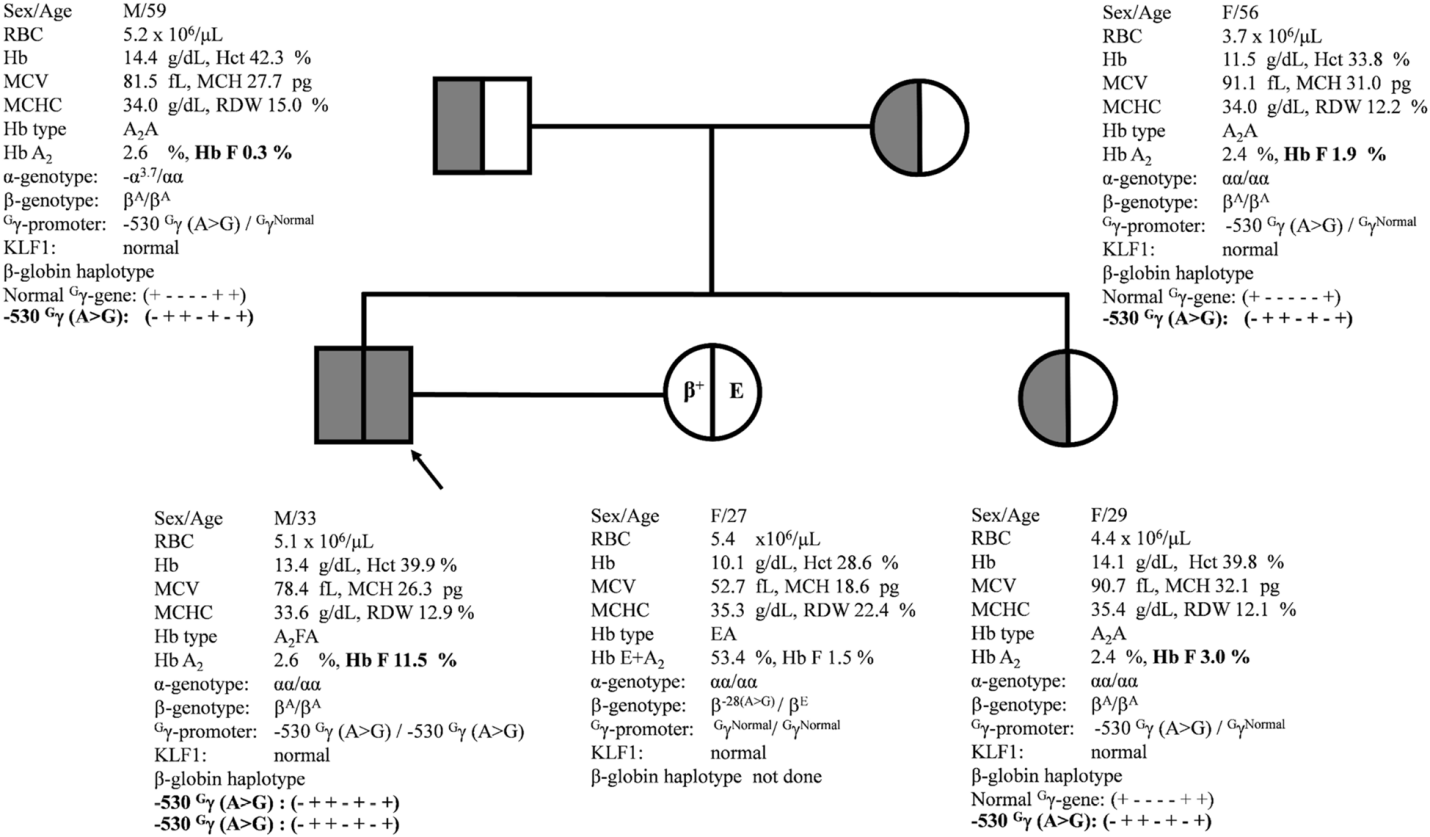
Pedigree analysis of a Thai family with the − 530 ^G^γ (A>G) HPFH. The arrow indicates the proband who was homozygous for the − 530 ^G^γ (A>G) whereas his parents and his sister were heterozygotes. The hematological parameters, Hb analysis, globin genotypes as well as β-globin gene haplotypes segregated in the family are presented.

Since the − 530 ^G^γ (A>G) novel mutation is located at the GATA-1 transcription factor binding motif of ^G^γ-globin gene promoter and is identified in homozygotic form, further family analysis was carried out as shown in Fig. 3. The proband was a 33-year-old male who was encountered with a mild hypochromic microcytosis without anemia (Hb 13.4 g/dL, MCV 78.4 fL and MCH 26.3 pg), and elevated Hb F level (11.5%) (case no. 20). Family analysis identified that his parents and younger sister were all carriers of HPFH with this novel − 530 ^G^γ (A>G) mutation. Apparently, heterozygosity for the − 530 ^G^γ (A>G) mutation is associated with normal hematological features and marginally elevated Hb F (1.9% in the mother and 3.0% in the sister). The father had alternatively reduced Hb F (0.3%) due to a co-inheritance of α^+^-thalassemia (3.7 kb deletion) since he was a double heterozygote for ^G^γ-HPFH and α^+^-thalassemia. In all members, the levels of Hb A_2_ were within normal range (2.4–2.6%). The proband’s wife (27-year-old) was a patient with Hb E-β^+^-thalassemia with mild hypochromic microcytic anemia with Hb 10.1 g/dL, MCV 52.7 fL and MCH 18.6 pg. Therefore, they were at risk of having a child with compound ^G^γ-HPFH/β^+^-thalassemia or ^G^γ-HPFH/Hb E syndrome. In addition, screening for KLF1 mutations previously described in Thai population^14^ yielded negative results in all members. β-Globin gene haplotype analysis on 7 polymorphic restriction sites within β-globin gene cluster as described in the “Materials and methods” section was carried out. Segregation of haplotypes in the family indicated that the − 530 ^G^γ (A>G) mutation was linked to the β-globin gene haplotype (- ++-+-+) in this Thai family and pointed to the same origin of this ^G^γ-HPFH in the father and the mother. Appropriate genetic counselling was provided to the family members.

## Discussion

In Thailand, we have extensively investigated the molecular basis of deletional form of HPFH and other high Hb F determinants including δβ^0^-thalassemia and ^A^γδβ^0^-thalassemia. At least 7 different deletional forms of HPFH and δβ^0^-thalassemia have been characterized in Thailand^5–10^. In contrast, the molecular basis of non-deletional HPFH in Thailand has not been investigated. This study represents the first extensive study of non-deletional HPFH among Thai population. At present, around 30 non-deletional HFPH mutations on both ^G^γ- and ^A^γ-globin genes have been characterized worldwide. These include 15 mutations each on ^G^γ-globin and ^A^γ-globin genes (Available at http://www.ithanet.eu/db/ithagenes, 2022 September, 25)^19^. We have now reported for the first time in Thai population, five different HPFH mutations found in 20 subjects including four ^A^γ-HPFH and a ^G^γ-HPFH. Of these five HPFH mutations, three known mutations were − 202 ^A^γ (C>T) (n = 3), − 196 ^A^γ (C>T) (n = 3) and − 158 ^A^γ (C>T) (n = 12). Two novel mutations namely − 117 ^A^γ (G>C) and − 530 ^G^γ (A>G) were unexpectedly detected. As shown in Table 1, because of the heterogeneity of hemoglobinopathies in Thai population, interactions of these non-deletional HFPH with α-thalassemia, β-thalassemia, Hb E, and KLF1 mutations were encountered in some cases.

The Black − 202 ^A^γ (C>T) and the Italian/Chinese − 196 ^A^γ (C>T), are located within the G-rich area upstream of the − 195 to − 202 of γ-globin gene. This DNA region is known to be the binding site of a ubiquitous transcription factor Sp1. As compared to the wild-type sequence, these two mutations decreased the binding of Sp1 on gel-shift experiment^20–22^. It has also been suggested that mutations in this G-rich sequence disrupt an intramolecular triplex proposed to be the binding site of a repressor, thereby increasing expression of γ-globin gene^23^. Carriers of the − 202 ^A^γ (C>T) and − 196 ^A^γ (C>T) reported previously had Hb F in the ranges of 1.6–3.9% and 12–21%, respectively^1,20–23^. In our study, the three carriers of the − 196 ^A^γ (C>T) had as expected the average Hb F of 14.3 ± 1.9% (Table 1). However, the Thai subjects with − 202 ^A^γ (C>T) demonstrated higher Hb F levels with the average of 9.9 ± 1.6% as compared to the previously reported cases, possibly due to the presence of Hb E and the − 158 ^G^γ *Xmn*I ( +) in all Thai subjects. In addition, two of them also carried the KLF1 mutations (H299D & T334R) (case no. 2 & 3), known to be associated with increased Hb F expression in Hb E syndrome^14^.

The − 158 ^A^γ (C>T) or the Cretan HPFH was originally described in three unrelated Greek adults with slightly increased Hb F level (2.9–5.1%) and normal hematological parameters. The − 158 ^A^γ (C>T) mutation has presumably resulted from a gene conversion event^1,24^. It is noteworthy that this form of HPFH was the most common one in Thai population, being detected in 12 of 20 Thai non-deletional HPFH subjects in this study (Table 1). Several explanations for the effect of this − 158 ^A^γ (C>T) mutation on Hb F production can be raised. We found that this mutation is associated in *cis* with the − 158 ^G^γ *Xmn*I ( +) polymorphism in Thai population. It has been noted that a 240-kDa activator protein, a member of the CAAT/enhancer-binding proteins family, binds to − 158 to -− 161 nucleotides of γ-globin gene promoter and induces expression of γ-globin gene. This may explain the effect of − 158 ^G^γ *Xmn*I (+) (C>T) and − 158 ^A^γ (C>T) to elevated γ-globin gene expression^25^. While the − 158 ^G^γ *Xmn*I (+) (C>T) polymorphism is associated with marginally elevated Hb F during erythropoietic stress, the − 158 ^A^γ (C>T) mutation is associated with higher Hb F expression with average of 7.8 ± 6.8% (Table 1). This likely be due to the fact that ^A^γ-globin gene is more effective in competition with ^G^γ-globin gene for the Locus Control Region (LCR) in the adult stage^25^. The combined action of − 158 ^A^γ (C>T) in *cis* with − 158 ^G^γ (C>T) may lead further to higher Hb F production than having − 158 ^G^γ (C>T) alone^24^. In addition, study in Chinese and Thai subjects has identified that the − 158 ^G^γ *Xmn*I (+) (C>T) was linked to the + 25 (G>A) polymorphism of ^A^γ-globin promoter (rs368698783), the binding motif of Ly-1 antibody reactive (LYAR) transcription factor. This polymorphism decreased the binding efficiency of the repressor of γ-globin genes leading to increased Hb F production^26^. We found that all Thai subjects with − 158 ^A^γ (C>T) HPFH mutation also carried the − 158 *Xmn*I ^G^γ (+) and the + 25 ^A^γ (G>A) polymorphism. This should explain the higher Hb F production in these Thai subjects (2.1–27.1%; mean ± SD: 7.8 ± 6.8%) found with the HPFH mutation alone or in combinations several common hemoglobinopathies including Hb E, β-thalassemia, α-thalassemia, and KLF1 mutation (G176Afs*179). The lowest Hb F of 2.1% was found in a heterozygous subject with the − 158 ^A^γ (C>T) in combination with Hb E and α^+^-thalassemia (3.7 kb deletion). This is not unexpected since it has been known that co-inheritance of α-thalassemia can lead to the reduced Hb F level in several hemoglobinopathies^1,3,5,27–29^. Of interest is the finding of subject with complex interaction of the − 158 ^A^γ (C>T), α^+^-thalassemia (3.7 kb deletion), and β^0^-thalassemia (β^17(AAG>TAG)^) (Table 1, case no. 17) who had increased Hb A_2_ (4.3%) and as high as 27.1% Hb F (Table 1). We have noted previously that β-thalassemia heterozygotes with or without α-thalassemia are associated with elevated Hb A_2_ to the diagnostic ranges of β-thalassemia heterozygote, but Hb F is not elevated (< 1–2%)^30^. This is in contrast with the deletional form of high Hb F determinants in which co-inheritance with β-thalassemia is associated with normal Hb A_2_ level^5^. Although diagnosis of a β-thalassemia heterozygote with this complex interaction seen in the case no. 17 is not altered due to elevated Hb A_2_, an unusually increased Hb F (27.1%) in heterozygous β-thalassemia, as seen in Thai subject, might be a good marker for a co-inheritance of HPFH in β-thalassemia, requiring further investigation. We recommend therefore to investigate all cases of heterozygous β-thalassemia with Hb F higher than 2% for further investigation of a possible co-inheritance of HPFH. This is a very important and useful information at genetic counselling since co-inheritance of HPFH can ameliorate the severity of β-thalassemia disease^1–3^. The last case in this group of HPFH with − 158 ^A^γ (C>T) mutation is an adult male encountered with a homozygous for this mutation who had 7.6% Hb F. He also carried the heterozygosity for KLF1 mutation (p.G176Afs*179), and α^+^-thalassemia (3.7 kb deletion). Homozygosity for a − 158 *Xmn*I ^G^γ (+) was not unexpectedly noted due to the linkage of these two mutations. Again, high Hb F with normal level of Hb A_2_ and other hematological parameters (Table 1) is a good marker in routine practice for further investigation of HPFH determinant.

Unlike other globin genes which contain only one CCAAT motif, γ-globin gene has duplicated CCAAT sequences. The proximal CCAAT motif is located at -88 nucleotide and the distal one is found at − 115 regions. The proximal CCAAT motif is corresponding to the CCAAT motif of β-globin gene in which mutations in this motif result in reduced β-globin gene expression and β-thalassemia pursue. In contrast, mutations in the distal CCAAT motif of γ-globin gene results in higher γ-globin expression and HPFH phenotype^3,15^. This indicates that the two CCAAT motifs of γ-globin gene function differently. At least three proteins present in erythroid cells bind to this distal CCAAT motif and its flanking regions including a ubiquitous CCAAT binding factor (CP1), CCAAT displacement protein (CDP), and an erythroid specific protein NFE1^15,31,32^. It has been shown that the Greek/Black/Sardinian − 117 ^A^γ (G>A) and Japanese − 114 ^G^γ (C>T) HPFH mutations slightly increased the binding of CP1 and CDP but reduced the binding of NFE1 to the distal CCAAT motif^15,31,32^. A novel mutation at the same region, [− 117 ^A^γ (G>C)] identified in Thai subject with Hb E heterozygote in this study was associated with 13.5% Hb F. It is conceivable that this Thai − 117 ^A^γ (G>C) HPFH mutation should behave similar mechanism with that of the Greek/Black/Sardinian − 117 ^A^γ (G>A) HPFH mutation. Prediction of transcription factors binding to the region using the TFBIND program^18^ in comparison between the wild-type promoter, − 117 ^A^γ (G>A) Greek/Black/Sardinian HPFH, − 117 ^A^γ (G>C) Thai HPFH and − 114 ^G^γ (C>T) Japanese HPFH was carried out as shown in Table 2. This revealed different similarity scores of the binding sites for CCAAT related transcription factors between the wild-type, and these HPFH mutations, especially the M00254; V$CAAT_01 (NNN**R**RCCAATSA) for the CCAAT box as shown in Table 2. As compared to the wild-type sequence with a score of 0.91076, the increased score was found for the Greek/Black/Sardinian − 117 ^A^γ (G>A) with score of 0.922056. The Thai − 117 ^A^γ (G>C) and the Japanese − 114 ^G^γ (C>T) had decreased scores of 0.824343 and 0.797797, respectively.

**Table 2:** Comparison of similarity score among the wild-type, − 117 ^A^γ (G>A), − 117 ^A^γ (G>C), and -114 ^A^γ (C>T) sequences using the online TFBIND program for prediction of CCAAT related transcription factors binding affinity.

AC ID from TRANSFAC R.3.4	Name of the binding factor	Consensus sequence	Wild type	− 117 ^A^γ (G>A)^31,32^	− 117 ^A^γ (G>C) (this study)	− 114 ^G^γ (C>T)^15^
M00254 V$CAAT_01	CCAAT box (cellular and viral CCAAT box)	NNNRRCCAATSA	0.91076	0.922056	0.824343	0.797797
M00104 V$CDPCR1_01	CDP CR1 (cut-like homeodomain protein)	NATCGATCGS	0.80267	0.806578	0.798437	< 0.77
M00185 V$NFY_Q6	NF-Y (nuclear factor Y (Y-box binding factor))	TRRCCAATSRN	0.939877	0.955036	0.922919	0.821172
M00159 V$CEBP_01	C/EBP (CCAAT/enhancer binding protein)	NNTKTGGWNANNN	< 0.87	0.888817	< 0.87	< 0.87
M00190 V$CEBP_Q2	C/EBP (CCAAT/enhancer binding factor)	NNNTTGCNNAANNN	0.843209	0.869341	0.821862	< 0.82
M00116 V$CEBPA_01	C/EBPalpha(CCAAT/enhancer binding protein alpha)	NNATTRCNNAANNN	0.851779	0.851779	< 0.80	0.829879
M00109 V$CEBPB_01	C/EBPbeta (CCAAT/enhancer binding protein beta)	RNRTKNNGMAAKNN	< 0.81	0.829371	< 0.81	< 0.81
M00201 V$CEBP_C	C/EBP (C/EBP binding site)	NGWNTKNKGYAAKNSAYA	< 0.80	0.801226	< 0.80	< 0.80
M00072 V$CP2_01	CP2	GCNMNAMCMAG	< 0.78	0.801887	< 0.78	< 0.78

S = C or G, W = A or T, R = A or G, Y = C or T, K = G or T, M = A or C, N = any base pair.

Nucleotides between − 675 to − 526 of ^G^γ-globin gene has been proposed as a negative regulatory element based on study in transiently transfected K562 cell^33^. The Iranian − 567 ^G^γ (T>G) HPFH mutation (HBG2:c.− 620 T>G) which changed a GATA-1 binding motif to GAGA sequence (AGATAA>AGAGAA) was associated with increased Hb F of 5.9% and 10.2% in two Iranian subjects^33^. The five bp deletion between − 533 to − 529 (-ATAAG) of ^G^γ globin gene (HBG2:c.− 582_− 586del ATAAG), located at the GATA-1 binding site was also associated with HPFH phenotype in an Indian family^34^. Therefore, alteration of GATA-1 and related repressor proteins bindings to the motif should result in up-regulation of γ-globin gene expression. In contrast, the Thai − 530 ^G^γ (A>G) HPFH mutation which is located at the same region of GATA-1 binding motif (AGATAA>AGATAG) may have less effect on ^G^γ-globin gene expression since it does not modify the GATA-1 binding site (A/T)GATA(A/G) at this position significantly. Therefore, as shown in Table 3, in *silico* analysis of GATA-1 related transcription factors binding of the Thai − 530 ^G^γ-HPFH using the online TFBIND program showed less changes on the similarity scores as compared to the wild-type sequence which contrasts with the − 533 to − 529 (-ATAAG) Indian HPFH mutation which reduced binding of all GATA-1 isoforms. As shown in the table, we found that the Thai HPFH mutation had slight increased binding scores of two GATA-1 isoforms and decreased binding scores for another two GATA-1 isoforms. The mechanisms underlying up-regulation of γ-globin gene in these HPFH mutations may be difference. Accordingly, the effect of the − 530 ^G^γ (A>G) Thai HPFH mutation on the ^G^γ-globin gene expression could be minimal. This might explain the marginal elevation of Hb F (1.9% and 3.0%) observed in pure heterozygotic form of the mother and the sister of the proband and indicated that two copies of the − 530 ^G^γ (A>G) mutation in homozygote state is required for dramatically increased in Hb F (11.5%) as seen in the proband (Fig. 3). These are only predicted in silico, combined with phenotypic expression observed in the family which may have limitation in prediction of the functional effect. Further functional study using transient expression in erythroid cell line and gel shift assay should provide more insight into a molecular mechanism of this novel variant^15,33^. It is noteworthy that the father who was heterozygous for the same mutation on the same chromosome background with β-haplotype (-++-+-+) and − 158 *Xmn*I ^G^γ (−) had normal Hb F level (0.3%) due to a co-inheritance of α^+^-thalassemia. It is evidenced from the Indian and Thai HPFH families that subjects with combined non-deletional HPFH mutation and β-thalassemia mutation in *trans* had elevated Hb A_2_ for β-thalassemia trait i.e., 3.6–3.9% in Indians^34^ and 4.3% in Thai (Table 1, case no 17). This is in contrast with combined deletional high Hb F determinants and β-thalassemia which are associated with normal levels of Hb A_2_^5^.

**Table 3:** Comparison of similarity score among the wild-type, − 530 ^G^γ (A>G), and − 533 to − 529 ^G^γ (-ATAAG) sequences using the online TFBIND program for prediction of GATA related transcription factors binding affinity.

AC ID from TRANSFAC R.3.4	Name of the binding factor	Consensus sequence	Wild type	− 530 ^G^γ (A>G) (this study)	− 533 to − 529 ^G^γ (-ATAAG)^33^
M00075 V$GATA1_01	GATA-1 (GATA-binding factor 1)	SNNGATNNNN	0.805035	0.870188	< 0.77
M00126 V$GATA1_02	GATA-1 (GATA-binding factor 1)	NNNNNGATANKGNN	0.863125	0.875312	< 0.77
M00127 V$GATA1_03	GATA-1 (GATA-binding factor 1)	RNSNNGATAANNGN	0.788829	< 0.78	< 0.78
M00128 V$GATA1_04	GATA-1 (GATA-binding factor 1)	NNCWGATARNNNN	0.865502	0.849877	< 0.81
M00076 V$GATA2_01	GATA-2 (GATA-2; GATA-box binding factor 2; GATA2; NF-E1b (chick))	NNNGATRNNN	0.853406	0.904826	< 0.78
M00077 V$GATA3_01	GATA-3 (GATA-binding factor 3)	NNGATARNG	0.887461	0.915817	< 0.82
M00203 V$GATA_C	GATA-X (GATA binding site)	NGATAAGNMNN	0.967071	0.924511	< 0.83

S = C or G, W = A or T, R = A or G, Y = C or T, K = G or T, M = A or C, N = any base pair.

In summary, three known and two novel HPFH mutations were identified for the first time in Thai population. This result indicates a diverse molecular heterogeneity of non-deletional HPFH in Thai population in addition to the deletional forms of δβ^0^-thalassemia, ^A^γδβ^0^-thalassemia, and HPFH described before^5–10^. Complex interactions between these non-deletional HPFH in both heterozygote and homozygote with other hemoglobinopathies commonly found in the region can lead to various hematological phenotypes and Hb F productions which could render diagnosis difficult. Identification of these non-deletional HPFH using rapid PCR diagnostic assays developed should improve this diagnosis in routine practice in the regions.

## Supplementary Information

Supplementary Information.

## Data Availability

All data generated or analyzed during this study are included in this published article.
