# Supplementary material for: Molecular basis of non-deletional HPFH in Thailand and identification of two novel mutations at the binding sites of CCAAT and GATA-1 transcription factors
Source: Sci Rep. 2023 Jul 24;13:11926. doi: 10.1038/s41598-023-39173-8 (PMC10366219; doi:10.1038/s41598-023-39173-8)
Supplement: Supplementary file 1 — Supplementary Information. [file 41598_2023_39173_MOESM1_ESM.doc]

**Supplementary Table S1 and Figures S1-S3** for …

Non-deletional HPFH in Thailand: Identification of two novel mutations at the binding sites of CCAAT and GATA-1 transcription factors

*By Singha K, et al.*

**Supplementary Table S1** Sequences and directions of oligonucleotide primers used in this study and their references

| **Primer** | **Direction** | **Sequence (5′→ 3′)** | **Reference** |
| --- | --- | --- | --- |
| F22 | Forward | TACTGCGCTGAAACTGTGGC | [9] |
| F35 | Forward | CCTGCACTGAAACTGTTGCT | This study |
| F38 | Reverse | TTGAGATAGTGTGGGGAAGGA | This study |
| F39 | Reverse | TTTGCATTGAGATAGTGTGGA | This study |
| F40 | Forward | AATACACATCATCGGGTGCC | This study |
| F41 | Reverse | GTTTCCAAATAAGAAGTGCTATACC | This study |
| γ4 | Forward | GGCCTAAAACCACAGAGA | [14] |
| γ5 | Reverse | CCAGAAGCGAGTGTGTGGAA | [14] |
| γ35 | Reverse | AGGTAGTTGTTCCCCTTCAA | [15] |
| G211 | Forward | GTTGGCCAGCCTTGCCTTC | This study |


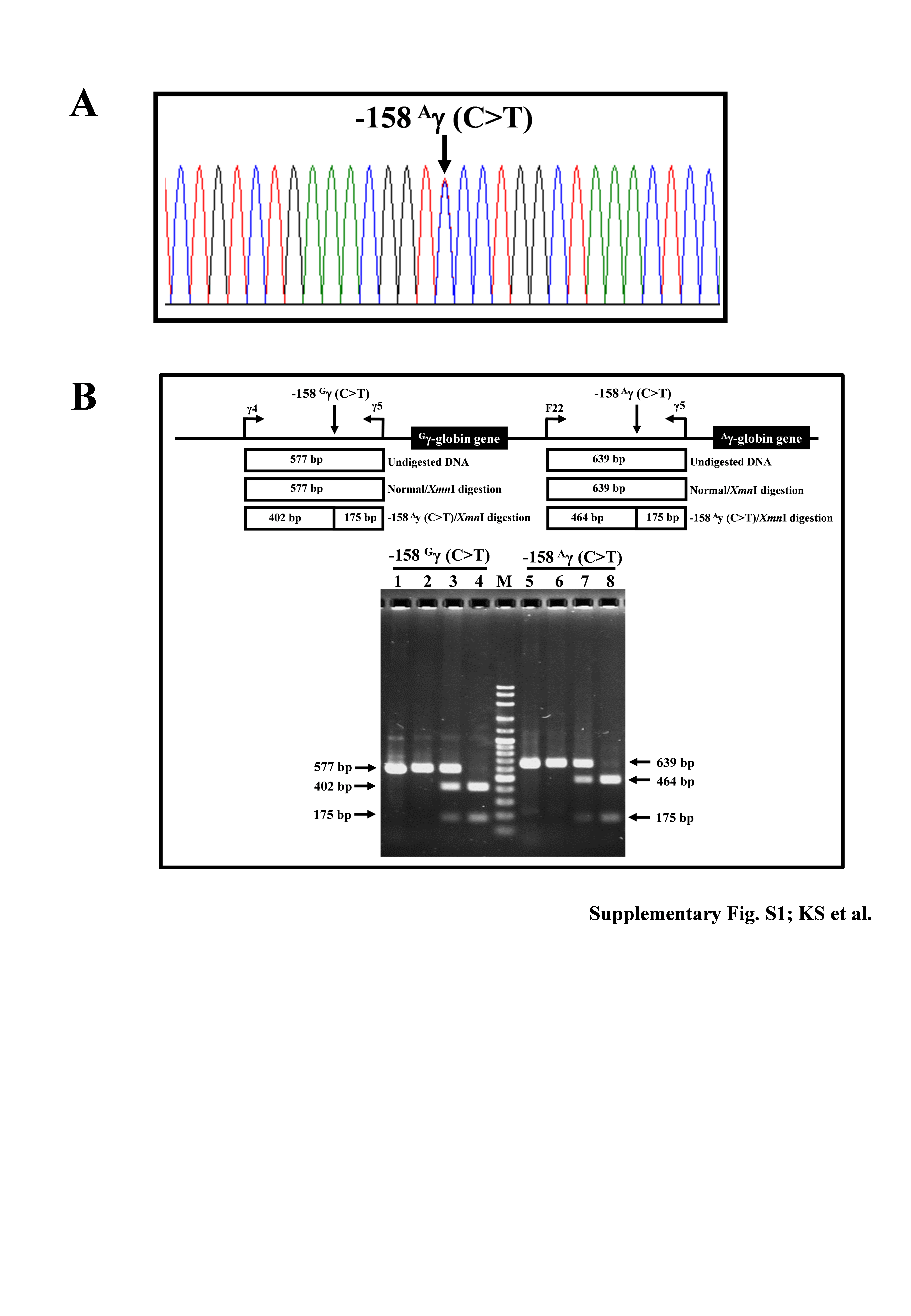


**Supplementary Fig. S1**

DNA sequencing profiles of homozygous -158 ^A^γ (C>T) (**A**) and identification of -158 ^G^γ (C>T) and -158 ^A^γ (C>T) using PCR-RFLP with *Xmn*I restriction enzyme (**B**). The locations and orientations of primer pairs (γ4 & γ5) and (F22 & γ5) specific for ^G^γ- and ^A^γ-globin promoters, respectively, and the sizes of amplified fragments are depicted. After digestion, the C allele [*Xmn*I (-)] derived fragment remains undigested in both ^G^γ- and ^A^γ-globin promoters, whereas the 577 bp of -158 ^G^γ-globin promoter with T allele [*Xmn*I (+)] derived fragment was digested into two fragments with 402 bp and 175 bp in lengths, and the 639 bp of -158 ^A^γ- globin promoter with T allele [*Xmn*I (+)] derived fragment was digested into two fragments with 464 bp and 175 bp in lengths. M represents the VC 100 bp plus DNA Ladder (Vivantis Technologies Sdn Bhd). Lane 1 is undigested amplified DNA for ^G^γ-globin promoter, lane 2 is *Xmn*I-digested amplified DNA of the -158 ^G^γ (C/C), lane 3 is *Xmn*I-digested amplified DNA of the -158 ^G^γ (C/T), lane 4 is *Xmn*I-digested amplified DNA of the -158 ^G^γ (T/T). For ^A^γ-globin promoter, lane 5 represents undigested amplified DNA, lanes 6-8 are *Xmn*I-digested amplified DNA of the -158 ^A^γ (C/C), -158 ^A^γ (C/T), -158 ^A^γ (T/T), respectively.


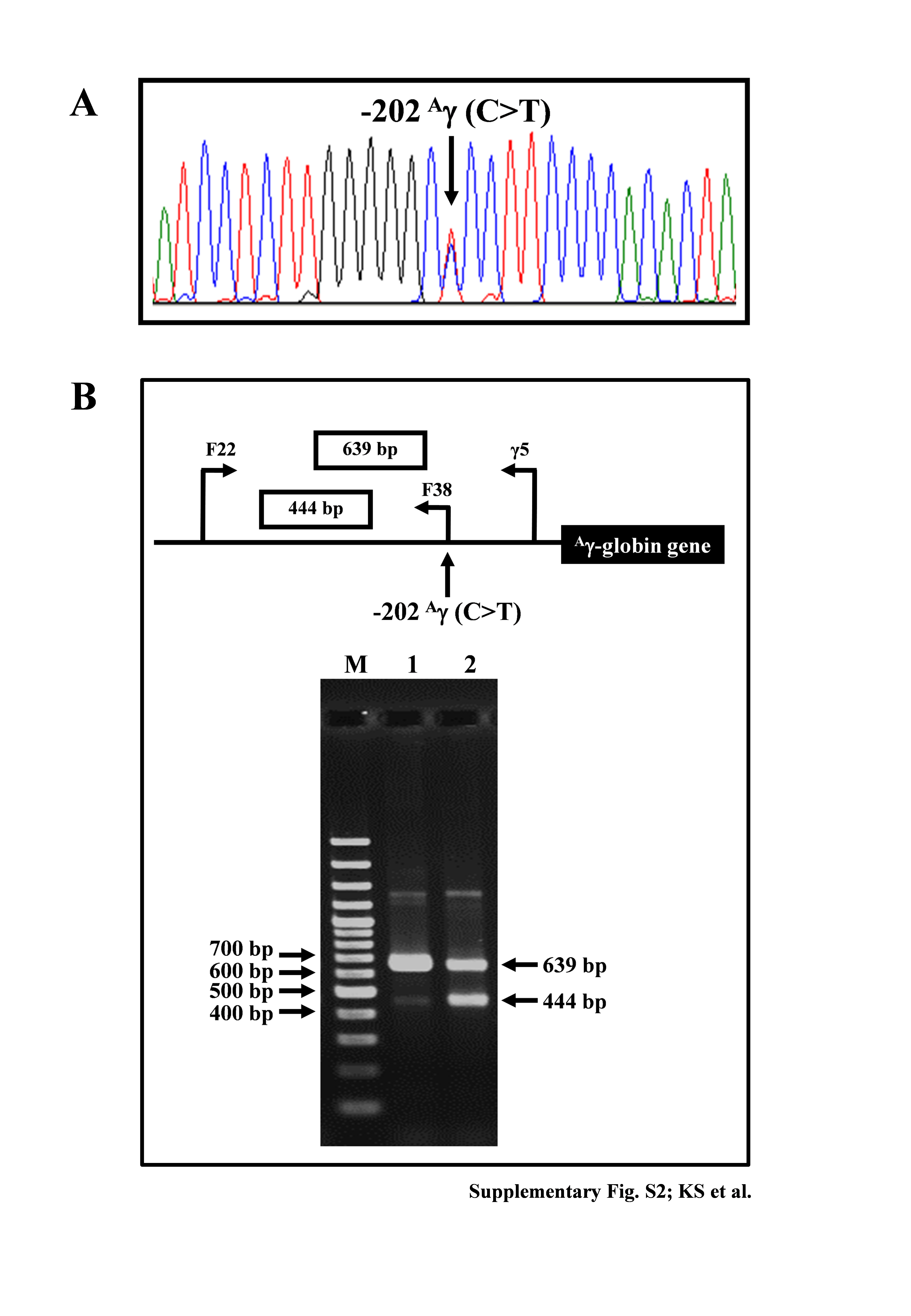


**Supplementary Fig. S2**

DNA sequencing profiles of heterozygous for -202 ^A^γ (C>T) (**A**) and identification of the -202 ^A^γ (C>T) by allele-specific PCR assay (**B**). M represents the VC 100 bp plus DNA Ladder. Lanes 1 and 2 are subjects with negative and positive for the mutation, respectively.


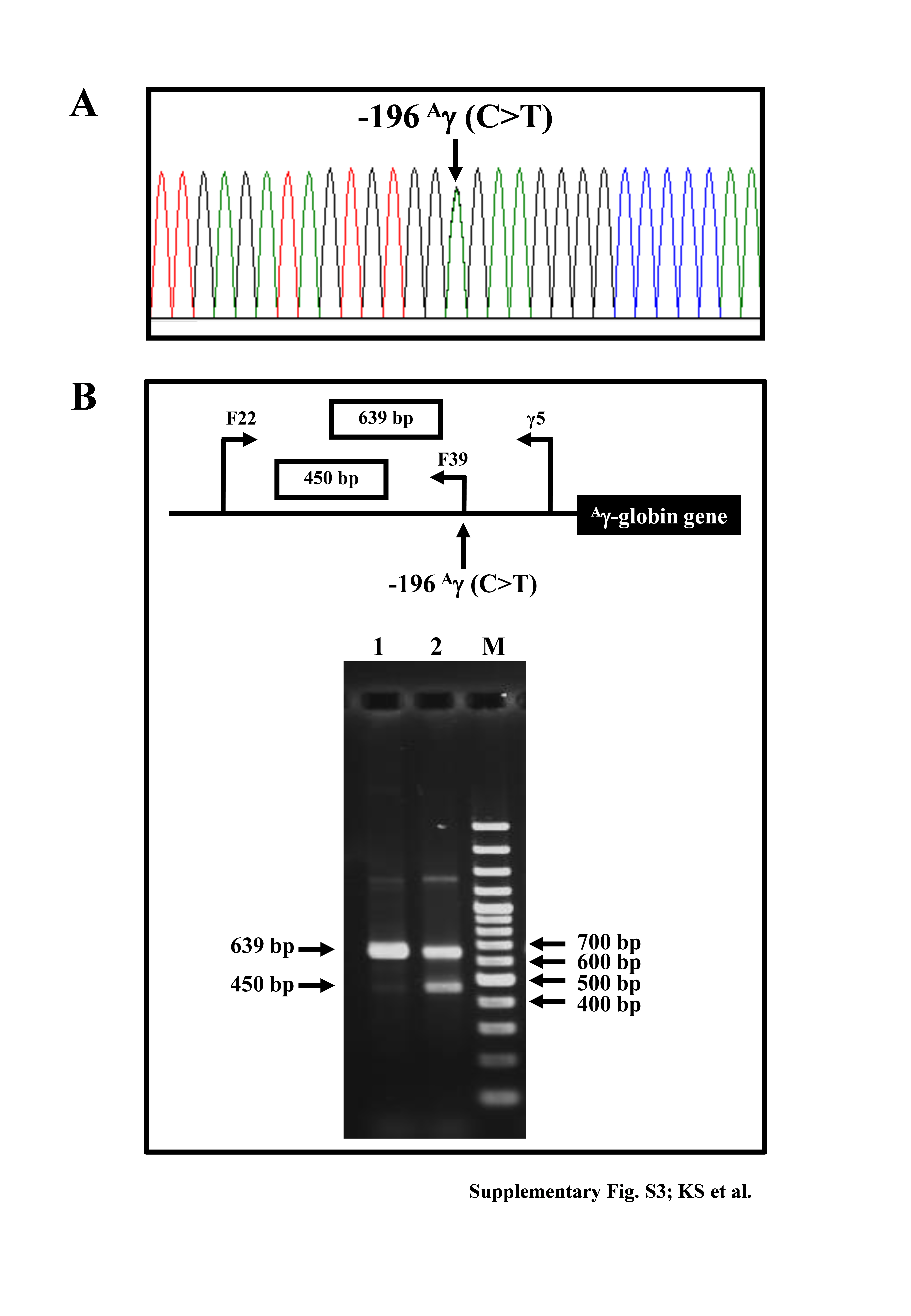


**Supplementary Fig. S3**

DNA sequencing profiles of heterozygous for -196 ^A^γ (C>T) (**A**) and identification of the -196 ^A^γ (C>T) by allele-specific PCR assay (**B**). M represents the VC 100 bp plus DNA Ladder. Lanes 1 and 2 are subjects with negative and positive for the mutation, respectively.
